# Lasing and Transport Properties of Poly[(9,9-dioctyl-2,7-divinylenefluorenylene)-alt-co-(2-methoxy-5-(2-ethylhexyloxy)-1,4-phenylene)] (POFP) for the Application of Diode-Pumped Organic Solid Lasers

**DOI:** 10.1186/s11671-017-2371-7

**Published:** 2017-11-22

**Authors:** Zhenyu Tang, Kunping Guo, Yulai Gao, Saihu Pan, Changfeng Si, Tao Xu, Bin Wei

**Affiliations:** 1 Key Laboratory of Advanced Display and System Applications, Ministry of Education, Shanghai University, 149 Yanchang Road, Shanghai, 200072 People’s Republic of China; 2 State Key Laboratory of Advanced Special Steel&School of Materials Science and Engineering&Laboratory for Microstructures, Shanghai University, Shanghai, 200072 People’s Republic of China; 3 School of Mechanical Engineering and Automation, Shanghai University, Shanghai, 200072 People’s Republic of China; 4 Laboratory for Microstructures, Shanghai University, Shanghai, 200072 People’s Republic of China

**Keywords:** Lasing materials, Transport properties, Diode-pumped lasers, Guided wave applications

## Abstract

This paper demonstrates the lasing and transport properties of a green conjugated polymer, namely POFP. High photoluminescence yields and excellent electron transport of POFP film make it promising for gain media. Low threshold value of 4.0 μJ/cm^2^ for amplified spontaneous emissions under a pulsed Nd:YAG laser at 355 nm was obtained, as well as a high Q-factor of 159. An inverted waveguide microcavity scheme has been developed to fabricate diode-pumped organic solid lasers (OSLs) using POFP. Gain narrowing with significant radiance increase was observed in the devices, giving evidence of the interference enhancement induced by microcavity and the lasing properties of POFP.

**Electronic supplementary material:**

The online version of this article (10.1186/s11671-017-2371-7) contains supplementary material, which is available to authorized users.

## Background

Organic semiconductors have attracted great interest in various applications of optoelectronic devices, such as organic light-emitting diodes (OLEDs) and organic photovoltaic (OPV) cells [1, 2], due to their advantages of mechanical flexibility, easy solution processing, and low-cost fabrication [3–5]. Among the organic semiconductor materials, conjugated polymers can be designed to have photoluminescence quantum yield (PLQY), large stimulated emission cross sections, and a wide emission range across the visible spectrum [6], which have triggered new research toward the possibility of using them as gain media for optical amplifiers and electrically pumped lasers [7, 8]. Since the realization of optically pumped organic solid lasers (OSLs) from polymers in 1996 [9], much efforts have been investigated to synthesize low threshold organic gain materials. Wenger et al. reported that organic laser device based on poly(9,9-dioctylfluoren-2, 7-diyl-alt-benzothiadiazole) (F8BT) showed a low lasing threshold of 6.1 μJ/cm^2^ [10]. Corrugated fluorene copolymers such as poly(phenylene vinylene) (PPV), polyfluorene (PF), and their derivatives present particular interest because of their semi-conductive and good fluorescent properties [11]. It is reported that such green- and red-emitting polymers have amplified spontaneous emission (ASE) thresholds ranging from 4.4 to 10.0 μJ/cm^2^ [4]. In this context, it is still desirable to develop novel organic gain media based on fluorine derivatives with extremely low thresholds and excellent lasing properties.

In addition to the new material development, various methods have been investigated to enhance the optical gain of polymers in OSLs. Femtosecond pulsed laser can be applied as a pumping source to obtain lower lasing thresholds [12], and two-dimensional distributed feedback (DFB) lasers were used to serve the same purpose [13]. For example, poly(2,5-bis(2′,5′-bis(2″-ethylhexyloxy)phenyl)-p-phenylenevinylene) (BBEHP-PPV) was used as the gain medium for OSLs based on a second-order DFB in Samuel’s group, to achieve thresholds near 1.2 μJ/cm^2^ [14]. Förster resonance energy transfer (FRET) is also an efficient technique, in which energy transfer takes place between a guest and a host material resulting in the increase of optical gain [15]. While these methods have already made a considerable success in improving optically pumped lasing, the electrical pumping has not, to date, proved successful in achieving gain or lasing. A primary challenge that obstructs the realization of electrically pumped OSLs is the limited current transmission capacity of organic materials. According to reports on the lasing threshold of optically pumped organic dye-doped films, the current density of ~ kA/cm^2^ is necessary for realizing population inversion of electrical pumping laser [16, 17]. Moreover, most precedent works were taking efforts in improving optical extraction by fabricating optical micro-resonator, which demanded complicated process and could hinder the carrier transport. As a result, it is necessary to develop a simplified microcavity scheme such as vertical feedback waveguide microcavity, which is easy to fabricate and can confine ASE in the active layer, resulting in spectral gain narrowing [18]. In addition, diode-pumped organic laser devices were proposed in our previous work as an alternative approach [19], in which an organic electroluminescent layer (EML) was used as a pumping source, while an organic laser dye layer acted as a high-efficient carrier transport layer and gain media.

In this work, the lasing properties of a green conjugated polymer, poly[(9,9-dioctyl-2,7-divinylenefluorenylene)-alt-co-(2-methoxy-5-(2-ethylhexyloxy)-1,4-phenylene)] (POFP), have been investigated. A low threshold of 4.0 μJ/cm^2^ for ASE with a high quality factor (Q-factor) of 159 were achieved for POFP thin films, indicating that it is easier to amplify by excitation with an extremely narrow ASE compared with other polymer dyes. The transport properties of POFP have been studied, showing that the use of POFP as electron transport layer could improve the efficiency of devices. Finally, an inverted structure with vertical microcavity was used to fabricate diode-pumped organic lasers, while POFP was applied as the optical gain media. It was found that the spectra of devices exhibited clear gain narrowing with significant radiance enhancement. The development of such material will be an interesting approach for future research on electrically pumped OSLs.

## Methods/Experimental

**Fig. 1:**
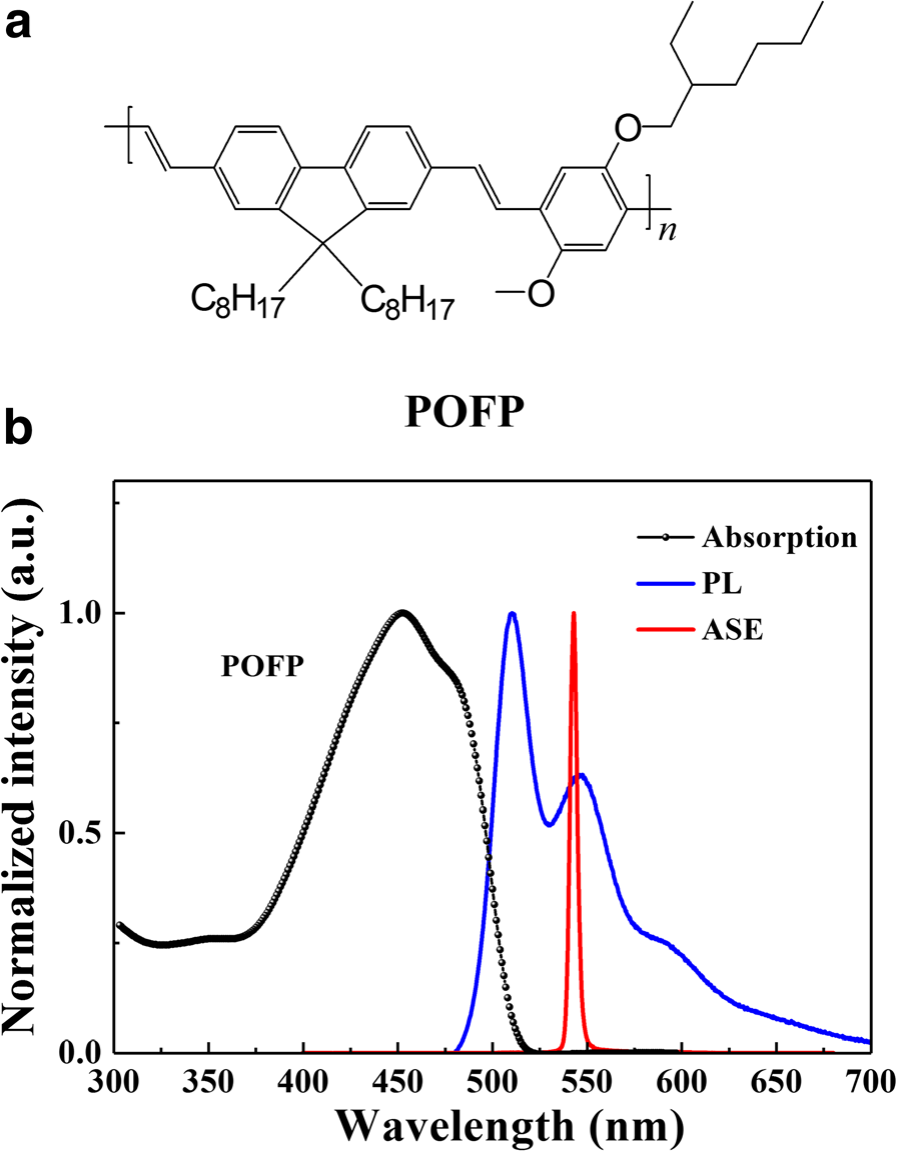
**a** The molecular structure of POFP. **b** The absorption, PL, and ASE spectra of POFP thin films

For this study, a green polymer POFP, which is a derivative in PPV family, was purchased from American H.W. SANDS. It is a pure substance with an average molecular mass that ranged from 40,000 to 80,000. The molecular structure is shown in Fig. 1a. The ASE and lasing properties of this conjugated polymer have not been reported before. POFP was dissolved in chloroform with a weight concentration of 0.7 wt%. The solution was spin-coated on the glass substrates to obtain POFP thin films with different thicknesses, followed by annealing at 60 °C for 20 min.

Hole-only and electron-only devices were fabricated to investigate the carrier transport properties of POFP. The structures of hole-only devices were as follows: device A: glass/ITO (180 nm)/POFP (75 nm)/NPB (5 nm)/Al (100 nm), and device B: glass/ITO (180 nm)/NPB (80 nm)/Al (100 nm). The architectures of electron-only devices were designed as: device C: glass/Ag (180 nm)/BCP (5 nm)/POFP (75 nm)/Al (100 nm) and device D: glass/Ag (180 nm)/BCP (5 nm)/Bphen (75 nm)/Al (100 nm). Here, N,N′-diphenyl-N,N′-bis(1-naphthyl)-1,1′-biphenyl-4,4″-diamine (NPB) was used as hole transportation layers, while 4,7-diphenyl-1,10-phenanthroline (Bphen) acted as an electron transportation layer. 2,9-Dimethyl-4,7 diphenyl-1,10-phenanthroline (BCP) was used as a hole-blocking layer. Finally, the diode-pumped OSLs with POFP film acting as gain media were demonstrated. Zinc sulfide (ZnS) was applied as an electron injection layer (EIL) for its efficient electron injection [20], while the molybdenum oxide (MoO_3_) acted as a hole injection layer (HIL). The device architectures were ITO/ZnS (2 nm)/POFP (150 nm)/AND:2wt%DSA-ph (10 nm)/NPB (10 nm)/2T-NATA (device E: 50 nm, device F: 125 nm)/MoO_3_ (5 nm)/Al (100 nm).

All devices were fabricated in conventional vacuum chamber by thermal evaporation of organic materials onto a clean glass substrate coated with an ITO (150 nm thick, 15 Ω per sheet) layer. Prior to use, the substrates were degreased in an ultrasonic bath by the following sequence: detergent, de-ionized water, acetone, isopropanol, and then cleaned in a UV-ozone chamber for 15 min. The typical deposition rates of organic materials, Ag, and Al, were 0.6, 0.1, and 5.0 Å/s, respectively. The device active area defined by the overlap between the electrodes was 4 mm^2^ in normal cases.

The ASE of POFP films were pumped by an Nd:YAG laser (FTSS 355-50, CryLaS) at an excitation wavelength of *λ* = 355 nm with a pulse width of about 1 ns and at a repetition rate of 100 Hz by focusing the excitation light with an irradiation area of 2.5 mm × 10 mm. A cylindrical lens and neutral density filters were used to adjust the excitation intensities. The emission radiation was collected from the edge of the film into an optical fiber connected to a spectrometer. The photoluminescence (PL) spectra were measured by using a FLSP 920 spectrometer series, while the absorption spectrum was recorded by a UV–vis spectrophotometer (U-3900H, Hitachi). The electroluminescence (EL) spectra of the devices were measured by a Photo Research PR-650 spectra scan spectrophotometer. The current–voltage characteristics were measured by a Keithley 2400 source-meter. The measurements were carried out in the dark at room temperature without device encapsulation.

## Results and Discussion

Figure 1b shows the absorption, PL, and ASE spectra of POFP thin films. POFP showed a strong emission in the green region which peaked at 512 nm with a shoulder at 550 nm, while the absorption peaked at 452 nm. The full width at half maximum (FWHM) of PL spectra was 60 nm. The ASE spectra of POFP pumped by an Nd:YAG laser at 355 nm showed a peak at 548 nm. Indeed, the strong absorption in the main blue region gives the possibility to pump POFP by using blue OLED.

**Fig. 2:**
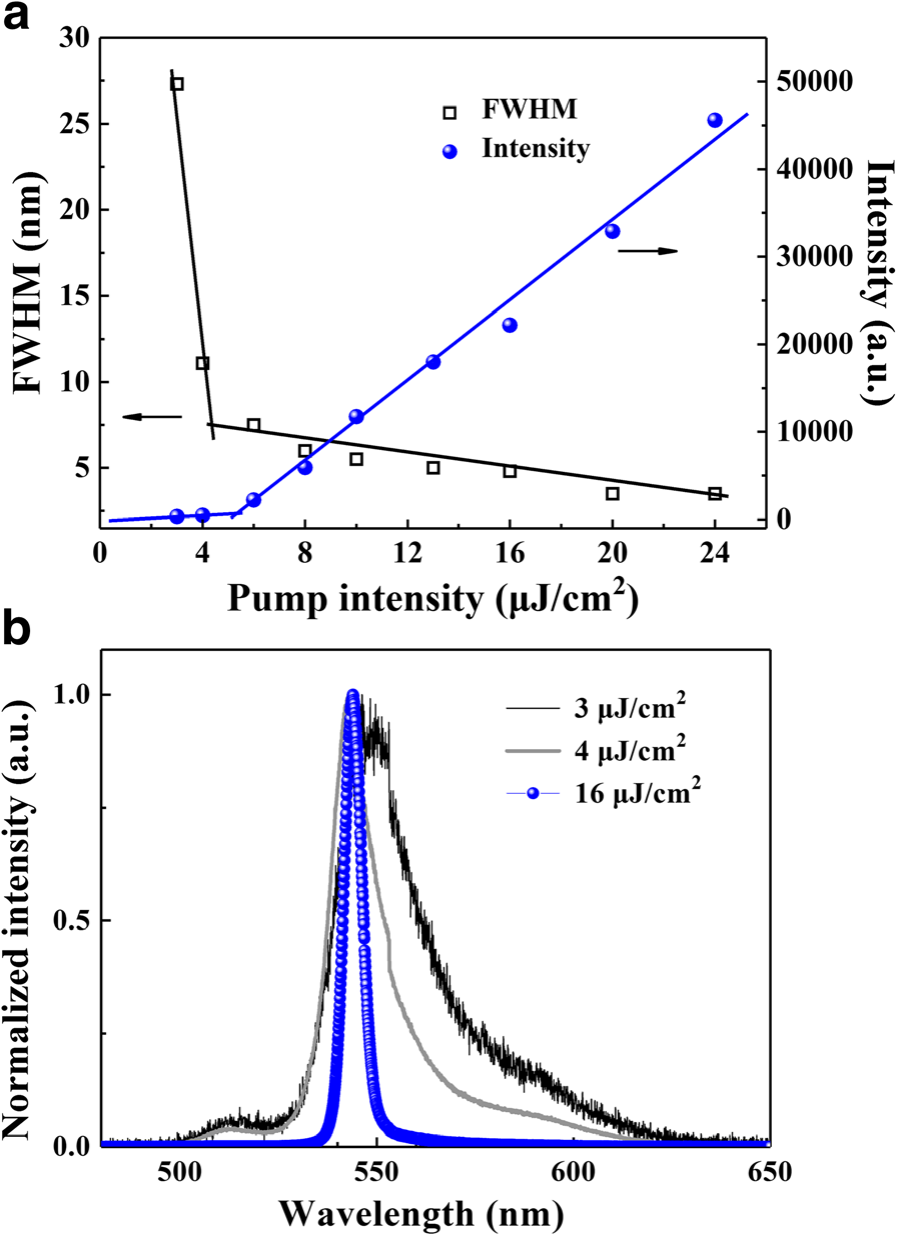
**a** Dependence of FWHM (squares) and peak intensity (spheres) of POFP films (135 nm) at various pump intensities. **b** The evolution of emission spectrum of POFP films (135 nm) with increasing pump intensity

Figure 2a shows the dependence of FWHM and the ASE output intensity of POFP films with a thickness of 135 nm at various pump intensities. When the pump intensity was increased from 1 to 20.0 μJ/cm^2^, the FWHM was found to decrease from 27.3 to 3.5 nm, while the ASE peak intensity was significantly amplified. The transition from linear to superlinear dependence of the ASE intensity as a function of pump intensity can be used as an indication of the ASE threshold. Furthermore, the value of FWHM was kept stable at a higher pump intensity, indicating the saturation state of ASE. The threshold energies of POFP films with different thicknesses from 60 to 165 nm were then measured, as it is summarized in Table 1. It was observed that the POFP film manifested a lowest threshold value of 4.0 μJ/cm^2^ with an optimal thickness of 135 nm. It is known that the pumping light cannot be effectively absorbed when the film is too thin; otherwise, the extinction would be induced by scattering in the case of thick film. Figure 2b shows the evolution of emission spectrum of POFP (135 nm) with increasing pump intensities of 3, 4, and 16 μJ/cm^2^. The gain narrowing of ASE spectra could be clearly observed.

**Table 1:** The relationship between POFP film thickness and threshold energy

POFP film thickness (nm)	60	70	110	125	135	165
Threshold energy (μJ/cm^2^)	11.2	7.5	6.4	6.0	4.0	8.4

Another important parameter to be considered is the Q factor, which describes the ability to retain light of any feedback structures. It can be used to evaluate the merits of ASE threshold in the model of Fabry-Perot resonators [21]. By calculation, the Q-factor of POFP is 159, which is a relatively high value compared with 109 for inorganic material CaF_2_ or Si [22] and 65 for pyrene-capped starburst polymer film [7].

**Fig. 3:**
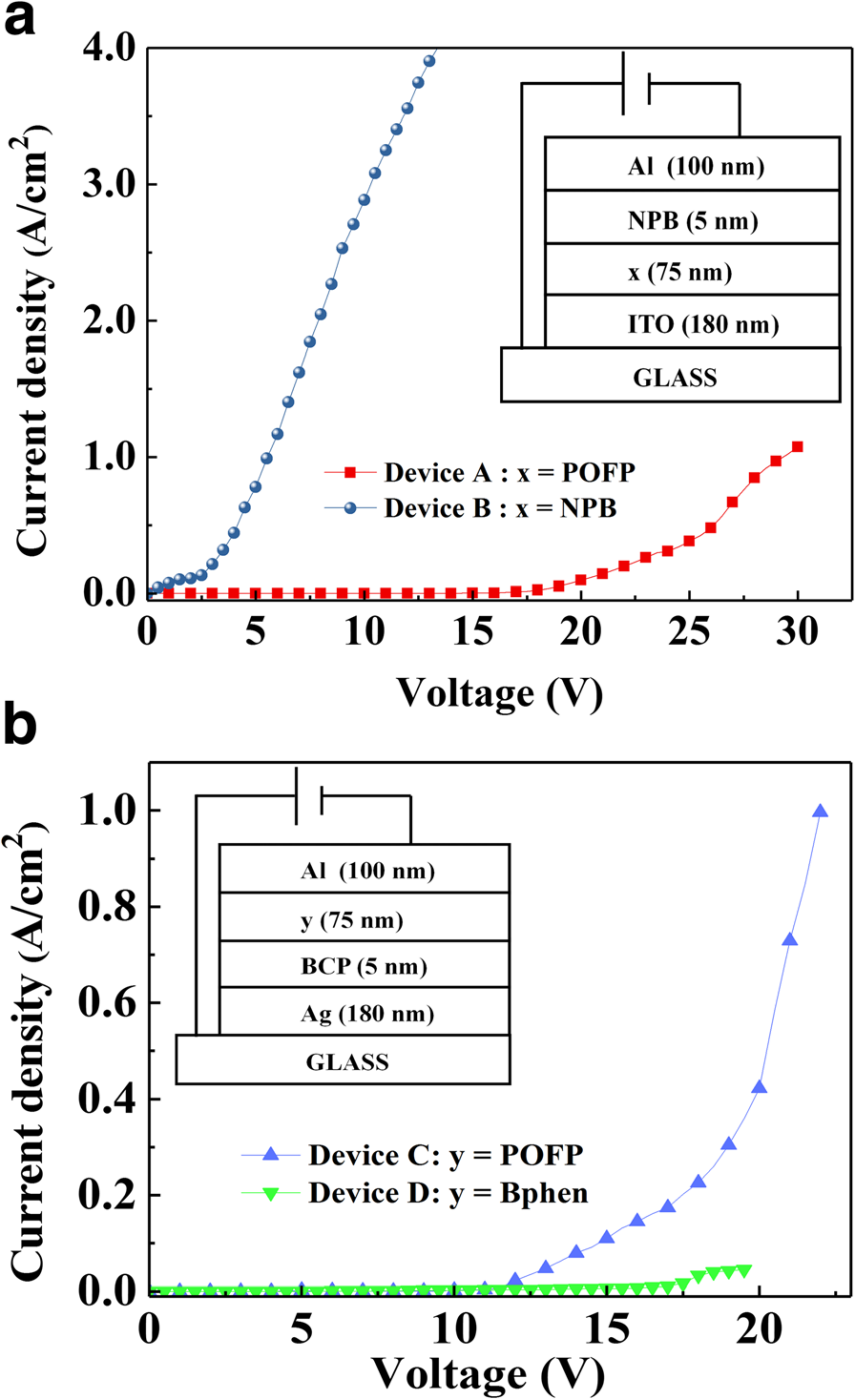
The *J*–*V* characteristics of **a** hole-only devices and **b** electron-only devices. The structures of the devices are shown in the insets

In order to fabricate diode-pumped OSLs with POFP, it is of great importance to understand its carrier transport characteristics. Two wildly used materials, NPB as a hole transport material and Bphen as an electron transport material, were employed to compare with the transport properties of POFP by means of single-carrier devices. As it is shown in Fig. 3a, device A and device B were fabricated to compare the hole transport characteristics between POFP and NPB. The *J*–*V* curves showed an obvious inferior hole transport ability of POFP. In contrary, the electron transport characteristic of POFP (device C) was measured to be better than that of Bphen (device D) as shown in Fig. 3b, indicating that POFP should work as an electron transport material in the OSLs.

**Fig. 4:**
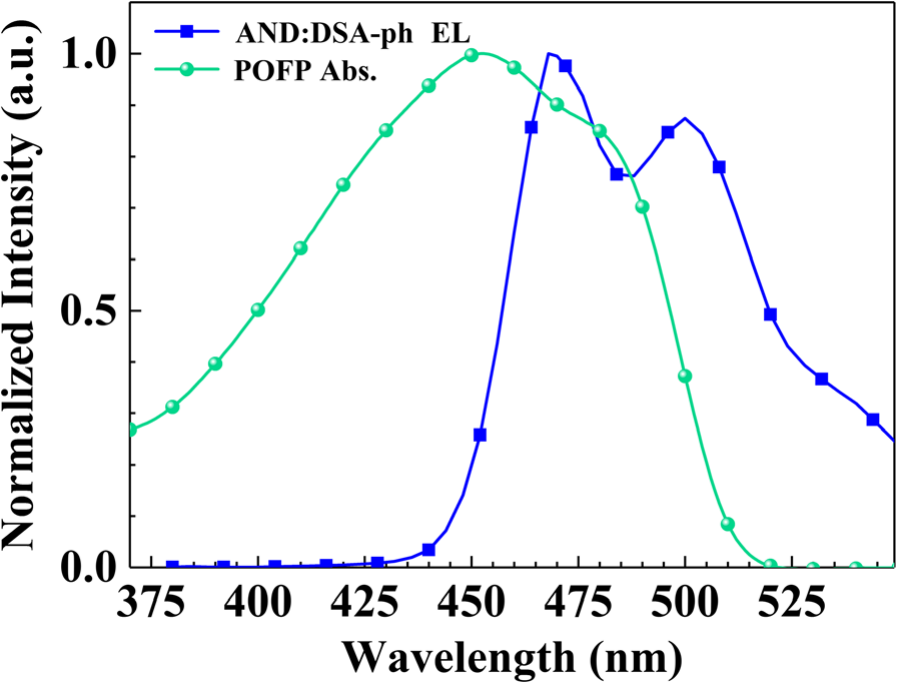
The EL spectrum of AND:2wt%DSA-ph and the absorbance spectrum of POFP

1,4-bis[N-(1-naphthyl)-N′-phenylamino]-4,4′-diamine/9,10-di(2-naphthyl) anthracene (AND) doped with blue dopant p-bis(p-N,N-diphenylaminostyryl)benzene (DSA-Ph) were chosen as the emitting layer (EML) in OSLs to pump POFP. Figure 4 shows the EL spectrum of AND:2wt%DSA-ph and the absorption spectrum of POFP. The EL spectrum of EML showed a peak at 468 nm, followed by a shoulder peak at 500 nm, exhibiting blue light emission. POFP was found to have high absorption at almost the whole blue region, making a wide range of overlap with the EL spectrum of EML, which offered the possibility of energy transfer to realize energy input from EML to gain media layer.

It is known that, in the microcavity devices, even a small reflection can make great effect on the device performance, which is due to the molecular films bounded between metal electrode and other reflector. Such structure can work as an optical resonator to determine the distribution modes of optical field and to modify the distribution of FWHM or luminous intensity. In order to use optical microcavity to obtain coherent light, one method is thin film interference theory. Based on the beam interferential theory, the relationship between optical path difference *δ* and phase difference *φ* is $\upvarphi =\frac{2\uppi}{\uplambda}\updelta$. When *δ* = mλ (*m* is the positive integer, stands for fringe order), it will form interference enhancement. When *δ* = (2m − 1)λ/2, there will be destructive interference. Considering the condition of interference enhancement in thin film system, the thickness of microcavity *d* should satisfy *d* = mλ/2, to produce feedback enhancement. Conversely, if the thickness *d* = (2m − 1)λ/4, the destructive interference will occur.

Based on this theory, devices of POFP pumped by EML with direct current (DC) were fabricated. The optical path difference should be *δ* = mλ, to generate interference enhancement, where *m* should be as low as 1 since the thickness of the film will affect the operation voltage of devices. In addition, the refractive of the film will have an influence over the wavelength, making λ^′^ = λ/n. Generally, the refractive index *n* of organic film is about 1.7. As a result, the minimum microcavity thickness *d*_c_ between metallic electrode and POFP film in order to achieve interference enhancement can be calculated as follows: ${d}_{\mathrm{c}}=\frac{\uplambda}{2n}=\frac{512\;\mathrm{nm}}{2\times 1.7}\approx 150\;\mathrm{nm}$. Similarly, the corresponding microcavity thickness to realize destructive interference was calculated to be 75 nm.

**Fig. 5:**
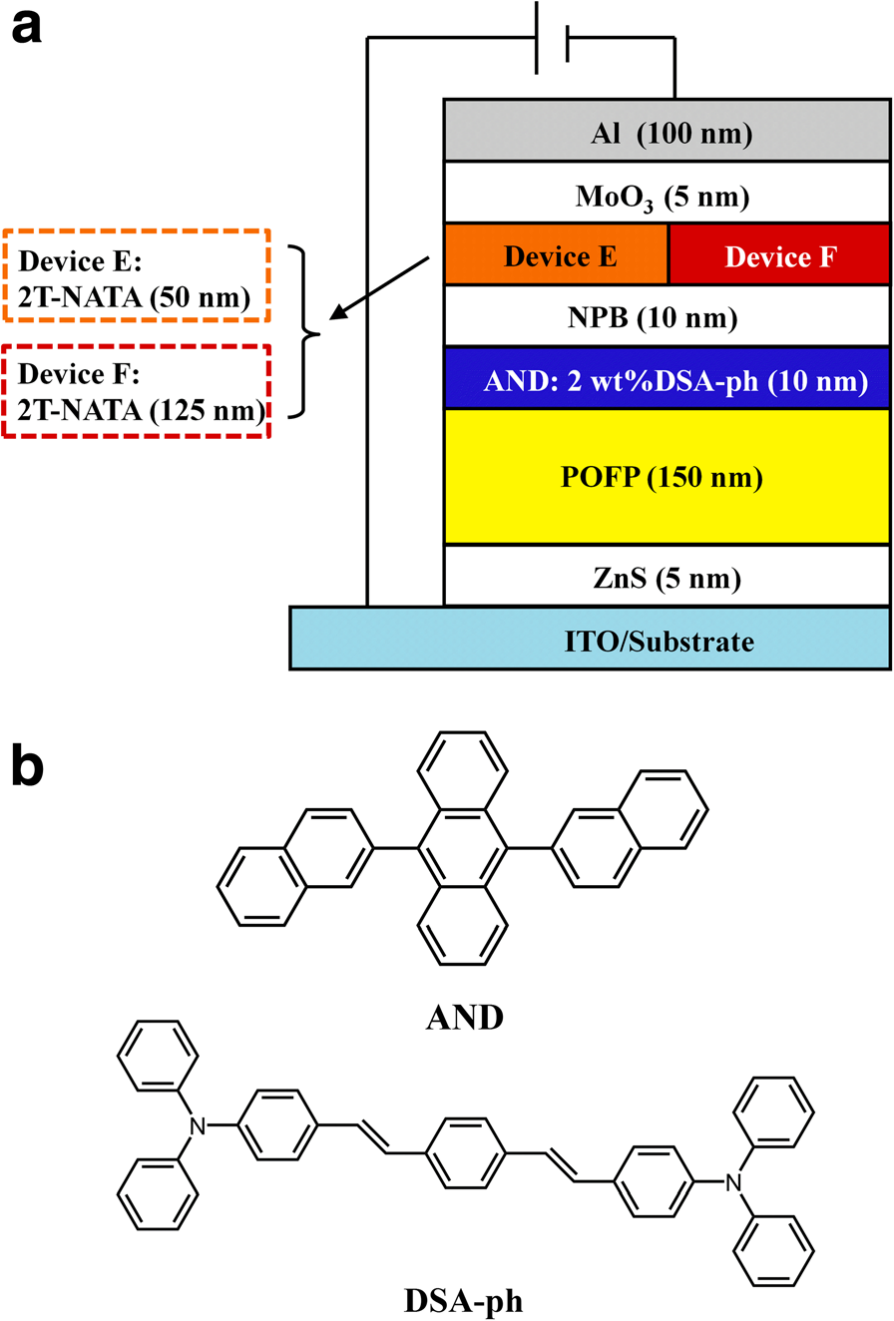
**a** Structures of diode-pumped OSL device E and device F. **b** Molecular structures of the emitting materials used in the devices

In this work, an inverted device structure was used to fabricate diode-pumped OSLs. We have recently found that the device structure of ITO/ZnS/Bphen/AND:DSA-ph/NPB/MoO3/Al could perform as extremely high-efficiency inverted OLEDs due to the formation of a favorable interfacial dipole layer at the metal sulfide-organic interface [20]. Furthermore, the inverted structure could also have great potential application for providing longer device lifetime because it can keep water and oxygen out from beneath sensitive electron injection materials [23]. Additionally, 2T-NATA was used to adjust the thickness of the microcavity. The device with destructive interference microcavity was fabricated as reference. The structures of invert devices (device E and device F) are shown in Fig. 5a, while Fig. 5b shows the molecular structures of the emitting materials.

The total thicknesses of MoO_3_/2T-NATA/NPB/AND:2wt%DSA-ph in the diode-pumped light-emitting devices were 75 and 150 nm for device E and device F, respectively, consistent with the calculated microcavity thickness. Electrons and holes could combine in the EML, emitting blue light, which will pump POFP and produce spontaneous radiation spectrum. Partial light can be subsequently reflected to POFP layer, while the light stimulated from POFP will finally cause interference with the reflected light to realize enhancement. The AND functioned herein as a host, while DSA-ph was the dopant. The influence of different doping concentrations (1.0, 2.0, and 5.0 wt%) and different dopants (DSA-ph and BCzVBi) on the performances of OSLs have been firstly investigated. It was found that the doping concentration of 2.0 wt% and the use of DSA-ph as dopant gave the optimized performance, as it is shown in Additional file 1: Figures S1 and S2 of the supporting information.

**Fig. 6:**
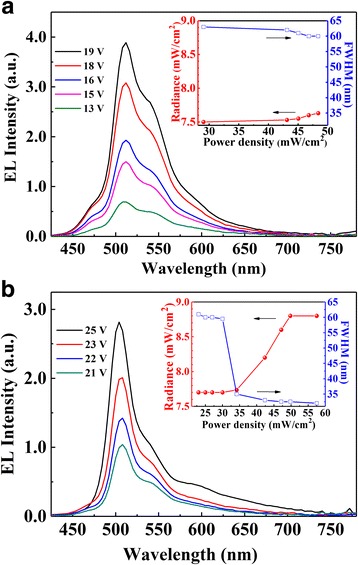
Evolution of EL spectra with increasing voltage of electrically pumped device E **a** and device F **b**. The insets show the dependences of radiance and FWHM at various power densities

Figure 6a, b shows the evolution of EL spectra with increasing voltage of diode-pumped device E and device F. The insets show the dependences of radiance and FWHM at various power densities. It can be found that the EL spectrum of both devices exhibited a peak at 512 nm with a shoulder, which was similar to the PL spectra of POFP, indicating that emergent light was from the excitation of POFP and stimulated by EML. In the insets of Fig. 6, it can be found that the FWHM of device F decreased from 60 to 32 nm with increasing of power density, while very slight narrowing of FWHM (from 62 to 60 nm) was observed in device E. Such phenomenon can be attributed to the destructive and enhanced interference induced by the calculated thicknesses of microcavities. Furthermore, the radiance of device F was significantly increased when the power density was higher than 34.0 W/cm^2^, but such enhancement was not found in device E. Typically, the narrowing of FWHM and the radiance enhancement could be considered as lasing characteristics; however, the FWHM of 32 nm was still too wide to be considered as lasing emission. In that case, the emission observed in device F with lasing properties can be attributed to waveguiding actions. It is known that waveguides are excellent spatial filters, light can emerge from the waveguide in a nearly diffraction-limited spot. Lighting can also resonantly leak into the substrate and then propagate next to the waveguide, giving a narrow emission [24]. In addition, luminescent microcavity is also considered as a structure which can induce emission with similar properties to laser. The local environment can affect strongly the spontaneous emission from a molecule, and wavelength-scale microstructures and microcavities can alter the spatial, spectral, and temporal properties of this light emission through interference effects, which can lead to narrow linewidths [21].

These results indicated that the emission measured in this work was not the electrically pumped lasing, but the spectrum narrowing and the increase of radiance can be attributed to lasing features, revealing the possibility to realize organic semiconductor lasers under diode pumping. Such results also demonstrated the great lasing characteristics and electrical performance of POFP as gain media. In addition, we have studied the influence of different polymer such as MEH-PPV on the performance of OSLs comparing with POFP (see Additional file 1: Supporting information, Figure S3). It turns out that POFP may be a more promising approach to the realization of organic electrically pumped laser devices in the future by using proper schemes, such as utilizing pulsed voltage to provide excitation-energy or introducing distributed Bragg resonance patterns on the substrate.

## Conclusions

In conclusion, we have investigated the photo-physical characteristics and electrical transport properties of an organic polymer laser dye, namely POFP. It was demonstrated that POFP exhibited an extremely low ASE threshold of 4.0 μJ/cm^2^ and a high Q-factor of 159, as well as a superior electron transport capacity compared with the commonly used ETL materials. In addition, POFP was used as gain media for diode-pumped OSLs, while an inverted structure with vertical waveguide microcavity has been developed to achieve interference enhancement. Lasing properties such as spectrum narrowing and radiance enhancement were observed in the devices, showing that it will be promising to apply POFP to organic electrically pumped semiconductor lasers.

## Additional file

Additional file 1: Supporting Information. (DOCX 1510 kb)

## Abbreviations

ASE: Amplified spontaneous emissions
DFB: Distributed feedback
EL: Electroluminescence
EML: Electroluminescent layer
FRET: Förster resonance energy transfer
FWHM: Full width at half maximum
OLEDs: Organic light-emitting diodes
OPV: Organic photovoltaic
OSL: Organic solid lasers
PL: Photoluminescence
PLQY: Photoluminescence quantum yield
Q-factor: Quality factor
