# Supplementary material for: Lasing and Transport Properties of Poly[(9,9-dioctyl-2,7-divinylenefluorenylene)-alt-co-(2-methoxy-5-(2-ethylhexyloxy)-1,4-phenylene)] (POFP) for the Application of Diode-Pumped Organic Solid Lasers
Source: Nanoscale Res Lett. 2017 Nov 22;12:602. doi: 10.1186/s11671-017-2371-7 (PMC5700016; doi:10.1186/s11671-017-2371-7)
Supplement: Additional file 1: — Supporting Information. (DOCX 1510 kb) [file 11671_2017_2371_MOESM1_ESM.docx]

**Supporting Information**

**Lasing and transport properties of poly [(9, 9-dioctyl-2,7-divinylenefluorenylene)-alt-co-(2-methoxy-5-(2-ethylhexyloxy)-1,4-phenylene)] (POFP) for the application of diode-pumped organic solid lasers**

By Zhenyu Tang,^1^ Kunping Guo,^1,3^ Yulai Gao,^2^ Saihu Pan,^3^ Changfeng Si,^1^ Tao Xu,^1,3,^* Bin Wei ^1,^*

*^1^* Key Laboratory of Advanced Display and System Applications

Ministry of Education, Shanghai University

149 Yanchang Road

Shanghai 200072 (P. R. China)

Correspondance: [xtld@shu.edu.cn](mailto:xtld@shu.edu.cn); [bwei@shu.edu.cn](mailto:zfyancat@upc.edu.cn)

*^2^* State Key Laboratory of Advanced Special Steel & School of Materials Science and Engineering & Laboratory for Microstructures

Shanghai University

Shanghai 200072 (P. R. China)

*^3^* School of Mechanical Engineering and Automation

Shanghai University

Shanghai 200072 (P. R. China)

The influence of the doping concentration to the performance of the OSLs has been studied in some previous works, for example: [Appl. Phys. Lett. 2004，85: 3301]. It is reported that the efficiency of Förster energy transfer from host to dopant could be confined when the doping concentration is too small or too high. It is thus important to optimize the DSA-Ph doping concentration. We have performed supplementary luminance-voltage characteristics of devices with different DSA-Ph doping concentrations, and EL spectra of each device at the same voltage. It was observed that optimum EL intensity and luminance were obtained at the DSA-Ph doping concentration of 2 wt%, as it is shown in Figure S1, indicating that the Förster energy transfer from the AND host to the DSA-Ph dopant is very efficient at this concentration. Such observation can justify the choice of 2 wt% DSA-Ph doping concentration used in this work.


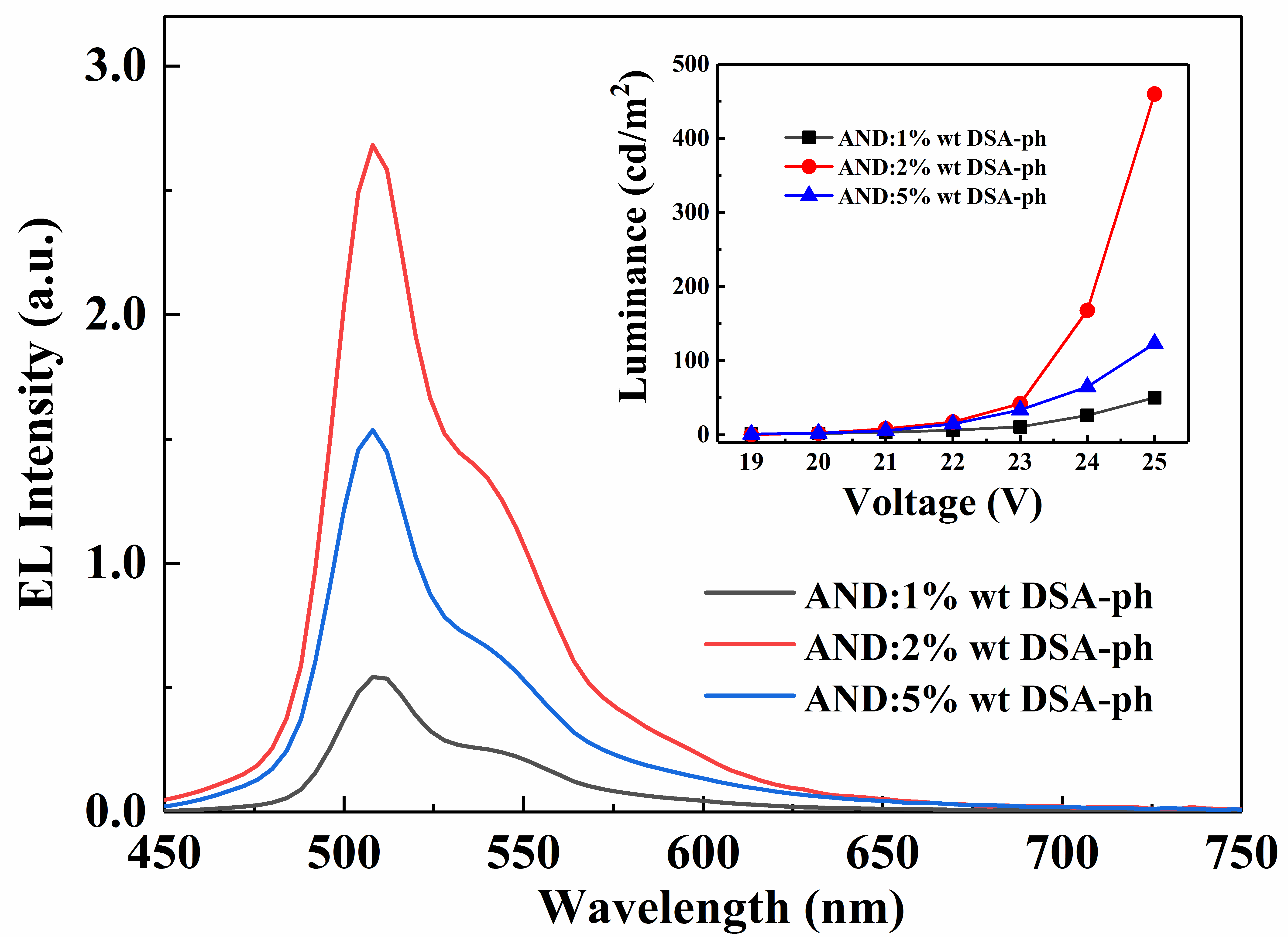


**Figure S1** EL spectra with different DSA-ph doping concentrations under 24 V. The inset shows the dependences of luminance and voltage at various doping concentrations.

Figure S2 (a) shows the LOMO/HOMO energy level diagram of BCzVBi, AND and DSA-Ph, which indicates that DSA-Ph can provide a more suitable path for the combination of electron and hole in the emission layer than BCzVBi. In addition, we have fabricated devices with BCzVBi and DSA-Ph as dopants. From the normalized EL spectra of Figure S2 (b), the device using DSA-ph as dopant gives clearly a better emission at 512 nm as well as a higher luminance intensity. Furthermore, the EL spectrum of BCzVBi shows an apparent shoulder peak, which means that the energy cannot transfer from host to dopant efficiently, resulting in quenching of exciton and low luminance.


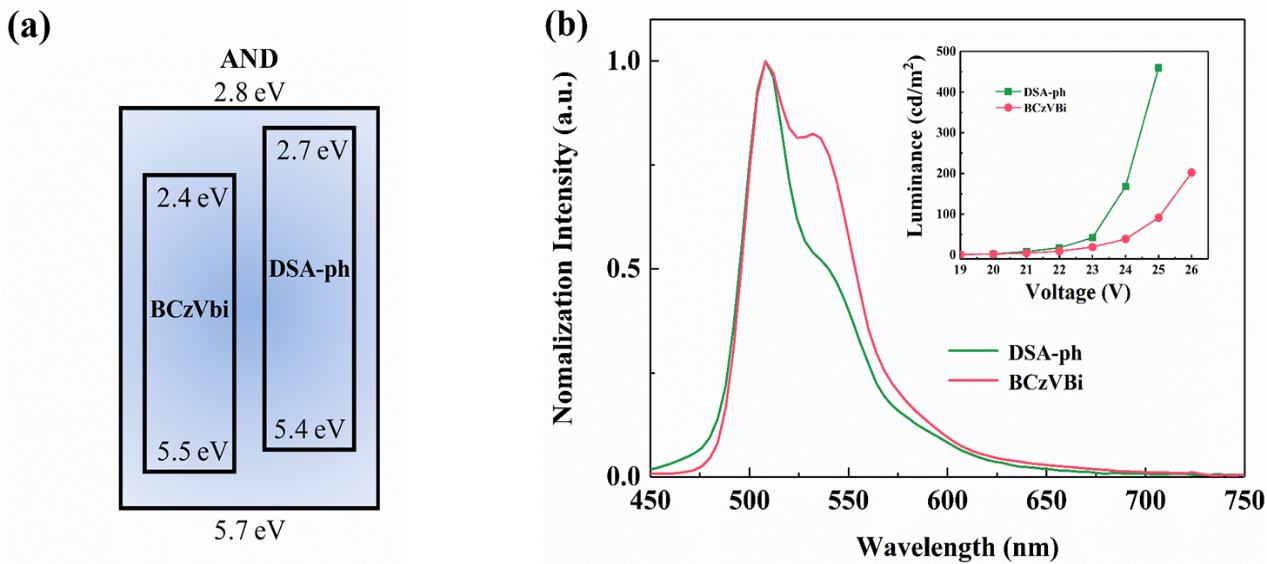


**Figure S2** (a) Energy level diagram of BCzVBi, AND and DSA-Ph, (b) Normalized EL spectra of using DSA-ph and BCzVBi as dopant. The inset shows the dependences of luminance and voltage.

We have studied another conjugated polymer MEH-PPV as a comparison of POFP. In the electrical pumped OSLs, the gain media should have not only low ASE threshold, but also good electrical properties to support energy transport in a very high density. MEH-PPV is a classical polymer luminescent materials with good hole transport properties [Journal of Physics D Applied Physics, 2010, 43 (20) :1503-1506]. The threshold pump power for ASE of MEH-PPV was reported as 6.0 kW/cm^2^ (62.5 μJ/cm^2^ ) [Synthetic Metals, 1999, 106 (1):35-38], which is much larger than that of POFP (4.0 μJ/cm^2^) in this work.

Two diode-pumped devices (Devices G and H) using MEH-PPV with destructive and enhanced interferences have been fabricated to make comparison with the ones using POFP in the same condition. After the calculations, the thickness of microcavity adapted for MEH-PPV should be 87.5 and 175 nm to achieve destructive and enhanced interferences, respectively. The structure of devices were designed as follows:

Device G: ITO/MEH-PPV(150 nm)/NPB(10 nm)/AND:2wt%DSA-ph(10 nm)/Bphen(10 nm)/Bphen:5 wt%CsCO3(67.5 nm)/Al

Device H: ITO/MEH-PPV(150 nm)/NPB(10 nm)/AND:2wt%DSA-ph(10 nm)/Bphen(10 nm)/Bphen:5 wt%CsCO3(155 nm)/Al

The Figure S3 (a) and (b) show the evolution of EL spectra with increasing voltage of diode-pumped Devices G and H. The insets show the dependences of radiance and FWHM at various power densities. It is known that the emission from MEH-PPV has a highest peak at 588 nm with a shoulder at 640 nm [Journal of Physics D Applied Physics, 2010, 43 (20) :1503-1506]. In Device G, the peak at 588 nm was found to be reduced because of the destructive interference, while it was amplified in Device H due to the interference enhancement. Moreover, another peak appeared at 468 nm, which was the emission of AND:DSA-ph. Because the MEH-PPV cannot absorb all the energy from the diode-pumping source under effect of interference enhancement, then the emission from AND:DSA-ph appeared.

More importantly, neither the narrowing of FWHM nor the radiance enhancement were observed in both devices using MEH-PPV, giving evidence of no lasing properties. As a result, it turns out that it is more promising to apply POFP in electrical-pumped OSLs comparing with MEH-PPV.


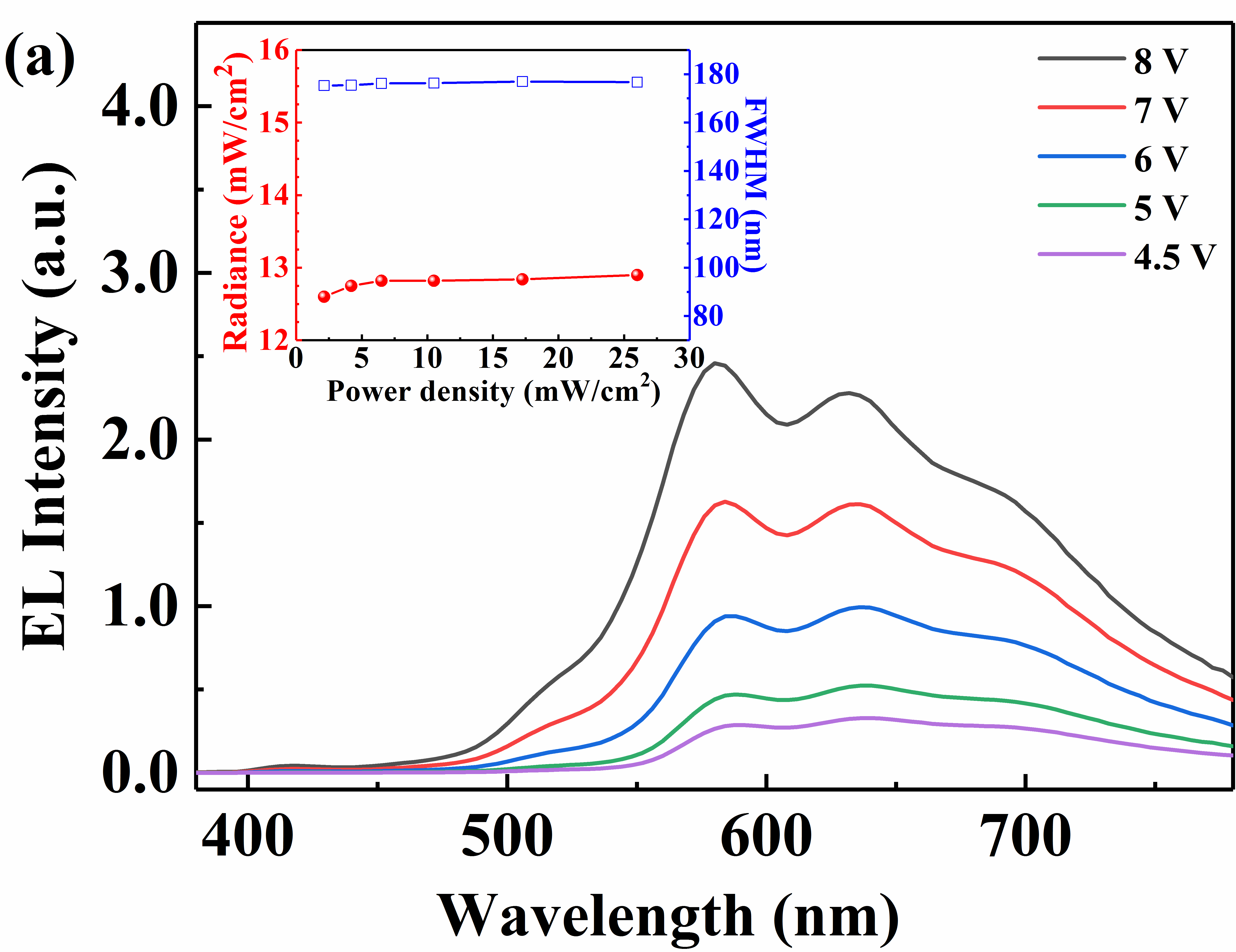


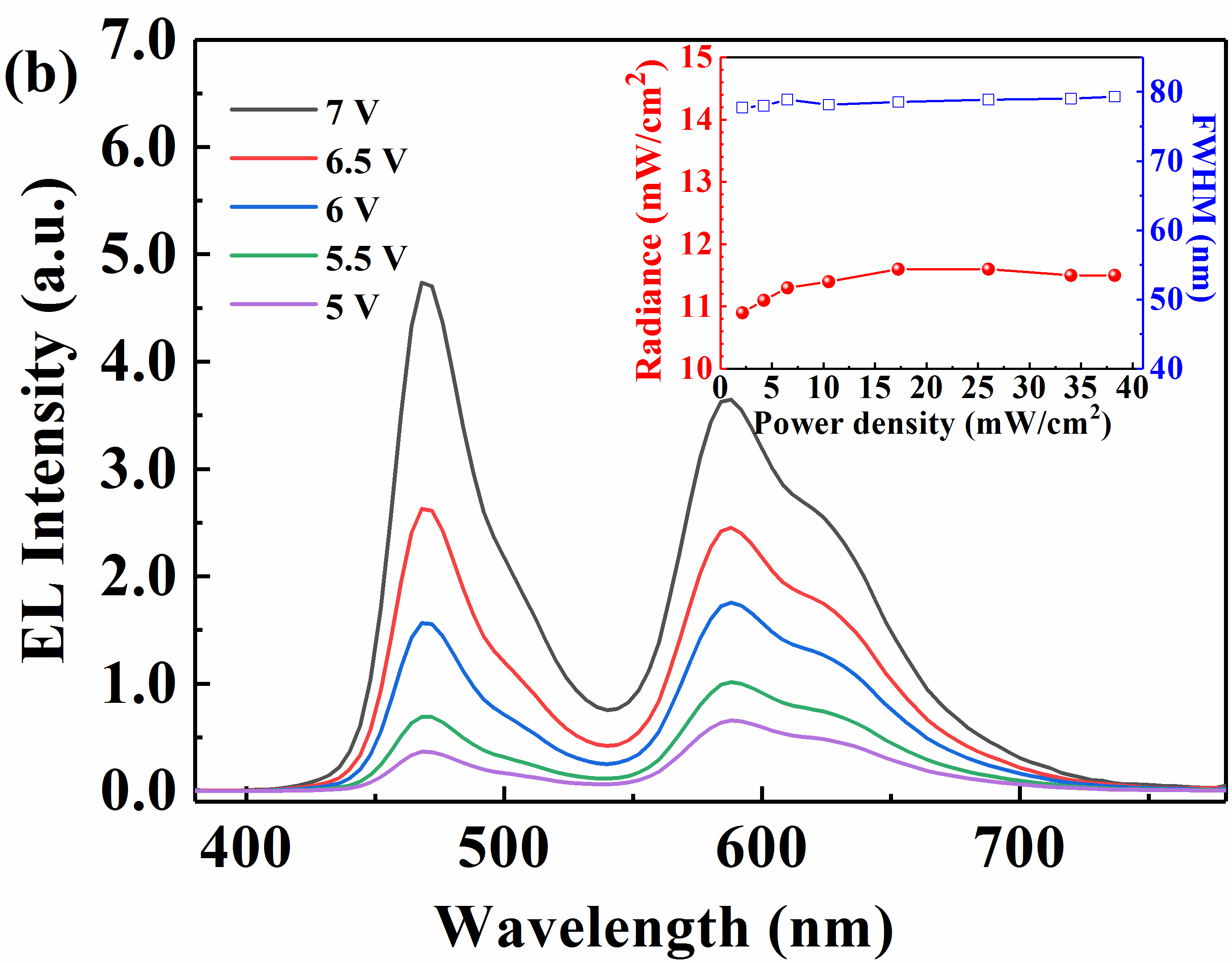


**Figure S3** Evolution of EL spectra with increasing voltage of electrical-pumped Device G (a) and Device H (b). The insets show the dependences of radiance and FWHM at various power densities.

**References**

S1:

Lee, Meng Ting, et al. Stable styrylamine-doped blue organic electroluminescent device based on 2-methyl-9,10-di(2-naphthyl)anthracene. Applied Physics Letters. 2004;85(15):3301-3303.

S3:

Zhu, Wenqing, et al. A very simple and chromatic-stable white organic light-emitting diode with a colour conversion hole injection layer based on solution process. Journal of Physics D Applied Physics. 2010;43(20):1503-1506.

Park, J. Y., et al. Amplified spontaneous emission from an MEH-PPV film in cylindrical geometry. Synthetic Metals. 1999; 106(1):35-38.
